# Rehabilitation for Cardiorenal Multimorbidity: Epidemiology, Functional Phenotypes, and Effects on Physical Function, Renal Trajectory, and Prognosis

**DOI:** 10.3390/jcm15072504

**Published:** 2026-03-25

**Authors:** Toshimi Sato, Masahiro Kohzuki

**Affiliations:** 1 Department of Physical Therapy, School of Health Sciences, Fukushima Medical University, Fukushima 960-8516, Japan; 2 Yamagata Prefectural University of Health Sciences, Yamagata 990-2212, Japan; kohzuki@yachts.ac.jp; 3 Tohoku University Graduate School of Medicine, Sendai, Japan

**Keywords:** cardiorenal syndrome, cardiovascular–kidney–metabolic syndrome, cardiac rehabilitation, multimorbidity, renal trajectory

## Abstract

**Background/Objectives:** Cardiac and renal dysfunction frequently coexist and interact bidirectionally, constituting cardiorenal syndrome (CRS). In aging societies, this overlap is increasingly conceptualized within cardiovascular–kidney–metabolic (CKM) syndrome, in which metabolic risk factors, chronic kidney disease (CKD), and cardiovascular disease cluster and worsen prognosis. Patients with cardiorenal multimorbidity exhibit reduced exercise tolerance, physical activity, and skeletal muscle function, leading to frailty, disability, recurrent hospitalization, and reduced tolerance of disease-modifying therapies. Although exercise-based rehabilitation is central to cardiovascular care and increasingly recognized in nephrology, its role in combined cardiac and renal dysfunction remains insufficiently integrated. **Methods:** This narrative review synthesizes cardiology and nephrology evidence using a functional framework. We address (i) the epidemiology and clinical significance of cardiorenal overlap across CRS/CKM, (ii) functional phenotypes defined by inactivity, low exercise capacity, sarcopenia/frailty, and disability, (iii) rehabilitation effects on physical function and renal trajectories, including renal endpoint validity (creatinine vs. cystatin C), and (iv) prognostic implications and evidence gaps. **Results:** Evidence from heart failure trials demonstrates that exercise-based cardiac rehabilitation improves health-related quality of life and reduces hospital admissions. In CKD, systematic reviews support exercise benefits for physical function and cardiometabolic risk. **Conclusions:** Although evidence remains limited, data support rehabilitation as a biologically plausible, function-centered therapeutic strategy.

## 1. Introduction

Cardiac and kidney diseases frequently coexist and exert bidirectional pathophysiologic effects that increase clinical vulnerability. These interactions were formalized in the American Heart Association (AHA) scientific statement on cardiorenal syndrome (CRS), which defines a spectrum of acute and chronic conditions in which dysfunction in one organ induces dysfunction in the other through hemodynamic, neurohormonal, and inflammatory mechanisms [1]. CRS emphasizes integrated physiology rather than isolated organ impairment, highlighting the central roles of venous congestion, reduced perfusion, neurohormonal activation, and iatrogenic influences in determining clinical trajectories. More recently, the cardiovascular–kidney–metabolic (CKM) syndrome framework expanded this concept by placing CRS within a broader multisystem context. The AHA CKM scientific statement emphasizes that metabolic risk factors, chronic kidney disease (CKD), and cardiovascular disease cluster and interact, accelerating adverse cardiovascular and renal outcomes [2]. A concise schematic comparison of the CRS and CKM frameworks is provided in Table 1 to highlight overlapping and distinct entry points for rehabilitation-oriented interventions.

Clinically, the burden of cardiorenal multimorbidity is often functional rather than solely biochemical. Reduced exercise tolerance, physical inactivity, skeletal muscle weakness, and frailty are common in patients with cardiovascular disease and CKD [3,4,5,6]. Among patients with combined cardiac and renal dysfunction, the severity of physical impairment strongly predicts survival [7]. Collectively, these functional impairments are clinically central in cardiorenal multimorbidity because they predict prognosis, constrain tolerance to disease-modifying therapies, and amplify vulnerability to hospitalization. Rehabilitation, defined as structured interventions targeting physical function, activity, and participation, directly addresses these impairments and aligns with the clinical needs of this population.

Exercise-based cardiac rehabilitation (CR) is a cornerstone of secondary prevention and heart failure management and is recommended in contemporary international guidelines [8,9]. Similarly, renal rehabilitation and structured exercise therapy are increasingly recognized in nephrology, supported by systematic reviews, consensus statements, and clinical practice guidelines [10,11,12]. However, implementation remains fragmented, and evidence specific to patients with concurrent cardiac and renal dysfunction is dispersed across cardiology and nephrology literature.

### Scope and Methodological Boundaries (Focused Narrative Review)

This focused narrative review synthesizes recent evidence on rehabilitation in cardiorenal overlap, focusing on outcomes relevant to patients and clinicians. We examine cardiovascular populations, including patients with heart failure and acute coronary syndromes, as well as kidney disease populations, including non-dialysis CKD, dialysis, and transplantation. Interventions include exercise-based CR, renal rehabilitation, and habitual physical activity. Primary outcomes include physical function (e.g., peak oxygen uptake [peak VO_2_], walking capacity, muscle strength, and health-related quality of life), renal markers (e.g., creatinine-based estimated glomerular filtration rate [eGFRcreat], cystatin C-based eGFR [eGFRcys], and albuminuria), and prognosis (e.g., hospitalization, major cardiovascular events, and mortality). When randomized evidence in cardiorenal overlap is limited, extrapolations from single-organ populations are clearly identified and interpreted cautiously.

To enhance transparency, we conducted a literature search of PubMed/MEDLINE, Scopus, and Web of Science for publications dated 1 January 2000 through 31 January 2026, using controlled vocabulary and free-text terms related to “cardiac rehabilitation,” “exercise,” “physical activity,” “chronic kidney disease,” “cardiorenal syndrome,” “cardiorenal overlap,” and “cardiovascular–kidney–metabolic.” Two reviewers independently screened records and assessed full texts, resolving disagreements by consensus. Clinical guidelines, systematic reviews, randomized trials, and observational cohort studies were prioritized, and mechanistic studies supporting biological plausibility were included where relevant. Evidence was synthesized qualitatively with explicit attention to renal endpoint interpretation. Pediatric studies, non-peer-reviewed items (e.g., conference abstracts), case reports or very small case series, and articles outside the defined scope were excluded.

This review integrates cardiology and nephrology evidence within a functional framework and is organized into four domains: (1) epidemiology and clinical relevance of cardiorenal overlap, (2) functional characteristics and phenotypes relevant to rehabilitation, (3) the effects of rehabilitation on physical function and renal trajectory, and (4) prognostic implications and future directions.

## 2. Epidemiology and Clinical Relevance of Cardiorenal Overlap

CRS is common among patients with heart failure and acute coronary syndromes, particularly in older adults with multiple comorbidities [1]. In cardiovascular disease, impaired kidney function is consistently associated with increased mortality, higher hospitalization rates, and reduced tolerance to guideline-directed medical therapy [8]. Comparative analyses of renal function decline before and after ischemic events suggest that renal deterioration may accelerate following cardiovascular disease onset [13,14,15,16,17]. Clinical trials and registry studies consistently identify kidney dysfunction as a major determinant of prognosis in cardiovascular populations [16,18,19,20,21,22]. In heart failure, kidney dysfunction during acute and chronic phases strongly predicts mortality [16,18].

In the VALIANT study, even mild reductions in estimated glomerular filtration rate were associated with increased mortality and cardiovascular events after acute myocardial infarction (AMI), highlighting kidney dysfunction as a critical prognostic modifier [19]. Similarly, data from the HIJAMI registry in Japan demonstrate progressively worse outcomes with increasing CKD severity and confirm CKD severity as an independent predictor of prognosis, regardless of successful coronary angioplasty [20,21]. Analyses from the CREDO-Kyoto AMI registry further show that early mortality after percutaneous coronary intervention is primarily cardiovascular, whereas later mortality is often non-cardiovascular; concomitant kidney dysfunction strongly predicts short- and long-term outcomes [22]. These findings underscore the importance of strategies aimed at preserving physical function and stabilizing renal trajectory in patients with cardiovascular disease.

Progressive CKD contributes to multiple adverse conditions, including impaired cardiovascular function, protein–energy wasting, metabolic acidosis, renal anemia, reduced bone strength, and cachexia, which collectively accelerate sarcopenia and frailty [23]. In patients with heart failure and coronary artery disease, CKD is consistently associated with reduced physical function [24,25,26]. Patients with combined cardiac and renal dysfunction therefore require systematic assessment and longitudinal monitoring of physical function and physical activity, both of which are closely linked to clinical trajectories.

## 3. Effects of Rehabilitation on Physical Function in Cardiorenal Populations

### 3.1. Evidence from CR

Exercise-based CR is supported by substantial evidence in patients with heart failure. A Cochrane systematic review demonstrated that, compared with no-exercise control, exercise-based CR improves health-related quality of life and reduces hospital admissions, although effects on all-cause mortality remain inconsistent across follow-up durations [27]. These findings are particularly relevant to patients with cardiorenal multimorbidity because hospitalization accelerates functional decline and worsens prognosis. CR is also being discussed across other cardiology domains beyond heart failure and ischemic heart disease. For example, a recent review summarized the potential roles of CR in atrial fibrillation, including functional and risk factor–oriented management within integrated care pathways. These examples highlight that CR models can be adapted to diverse cardiovascular phenotypes, providing a useful template for designing integrated cardiorenal rehabilitation programs [28].

Evidence specifically evaluating CR in cardiovascular patients with CKD remains limited. Hamazaki et al. reported that CR improves muscle strength and walking capacity across CKD stages, although severe kidney dysfunction may attenuate the magnitude of benefit [29]. Despite these favorable effects, patients with CKD remain underrepresented in CR trials, and referral and participation rates are low [30].

### 3.2. Evidence from Renal Rehabilitation

In patients with CKD, systematic reviews and meta-analyses consistently demonstrate that exercise training improves physical fitness, muscle strength, and blood pressure [10,31,32,33]. In advanced CKD and dialysis populations, structured exercise, including intradialytic programs, improves physical function and health-related quality of life, and some studies have examined survival-related outcomes [34,35,36]. Similarly, in patients with kidney transplantation, systematic reviews and randomized trials report improvements in physical function and selected domains of health-related quality of life following exercise interventions [37,38,39,40,41]. Collectively, these findings support rehabilitation throughout the continuum of kidney disease, although heterogeneity in study design and outcome definitions limits direct comparisons.

## 4. CR and Renal Outcomes

### 4.1. Center-Based CR and Renal Trajectory

Recent clinical studies—predominantly small, non-randomized cohorts—report that participation in CR is associated with favorable trajectories of renal markers in selected cardiovascular populations (Table 2) [42,43,44,45,46,47,48,49,50]. Takaya et al. demonstrated that 3 months of outpatient late-phase II CR in patients with AMI, with and without CKD, improved peak VO_2_ and reduced BNP levels in both groups. Notably, eGFRcreat improved in patients with CKD and remained stable in those without CKD, suggesting that participation in CR was associated with improvement or maintenance of renal function [43]. Similarly, Kimura et al. reported that 6 months of supervised outpatient CR was associated with a reduction in the urine albumin/creatinine ratio and maintenance of eGFRcreat, whereas patients without CR exhibited declining eGFRcreat and no change in albumin/creatinine ratio [44].

**Figure 1:**
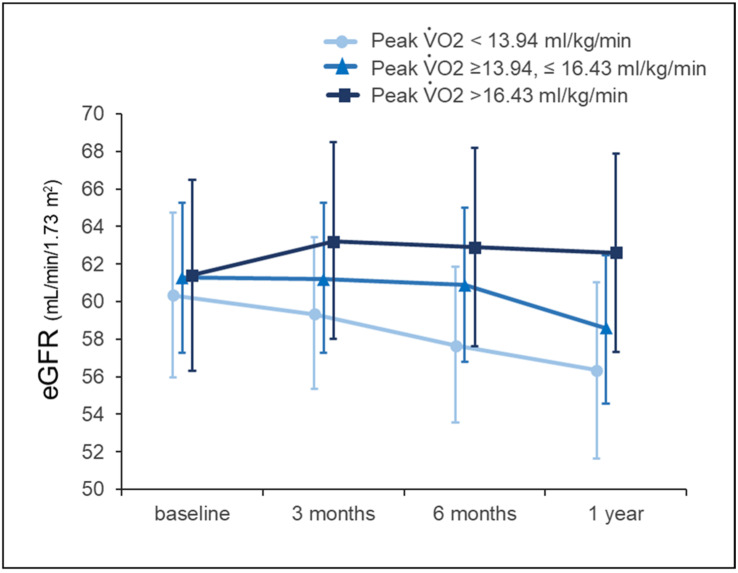
Longitudinal change in eGFR over 1 year after acute myocardial infarction (based on [50]). Data are presented as estimated marginal means, and error bar represents 95% confidence interval. eGFR, estimated glomerular filtration rate.

Toyama et al. [42] found that a 12-week exercise program in patients with cardiovascular disease and CKD improved eGFRcreat. Changes in eGFR correlated with improvements in anaerobic threshold and lipid profiles, including increased high-density lipoprotein cholesterol and reduced triglycerides, suggesting that exercise-related metabolic adaptations may be associated with changes in renal trajectory. In our observational study, higher peak VO_2_ achieved after CR independently predicted a more favorable 1-year renal trajectory (Figure 1) [51], supporting the role of exercise capacity as a determinant of kidney function trajectory.

The effects of CR on renal function in frailer patients, such as older adults, remain an important clinical question. Sasamoto et al. reported that 3 months of late-phase II CR in cardiovascular patients aged ≥ 75 years attenuated decline in eGFRcys, including among patients with CKD [49]. Rehospitalization rates during the intervention period were lower in the CR participation group (6.7%) than in the non-participation group (16.9%), suggesting that CR participation may be associated with maintenance of kidney function and reduced rehospitalization risk in older cardiovascular patients who are more susceptible to age-related renal decline. In contrast, Iso et al. reported that these associations may be attenuated with advancing age, with younger patients demonstrating greater benefit [45]. This finding aligns with geriatric principles indicating that biologic reserve and baseline functional capacity influence responsiveness to rehabilitation [52].

Long-term observational studies further support the association between CR participation and renal function trajectory. Fujimi et al. reported stable eGFR over 1 year of CR, with improvement observed among patients with lower baseline eGFR, particularly those with mild-to-moderate CKD [46]. Similarly, Kitajima et al. reported maintenance of eGFR and cardiopulmonary function over follow-up periods extending to 5 years in older cardiovascular patients [48]. These findings suggest that long-term CR may be associated primarily with stabilization of renal function rather than large short-term increases in filtration measures.

However, interpretation of Table 2 requires caution. Most available studies evaluating renal markers during CR are observational and include relatively small samples, often from single-country cohorts (notably Japan). Renal outcomes are typically based on surrogate markers (eGFRcys or eGFRcreat and albuminuria) over short follow-up durations, whereas hard renal endpoints have not been tested in large randomized trials in this context. Accordingly, the current literature supports hypothesis-generating signals of renal marker stabilization in selected participants rather than definitive renoprotection, and findings may be influenced by confounding (e.g., referral or selection bias and baseline functional reserve). Additionally, from a translational perspective, replication across diverse healthcare systems and rehabilitation delivery models is needed to determine whether these associations persist beyond single-country contexts.

### 4.2. Management of Habitual Physical Activity and Renal Trajectory in Cardiovascular Patients

**Figure 2:**
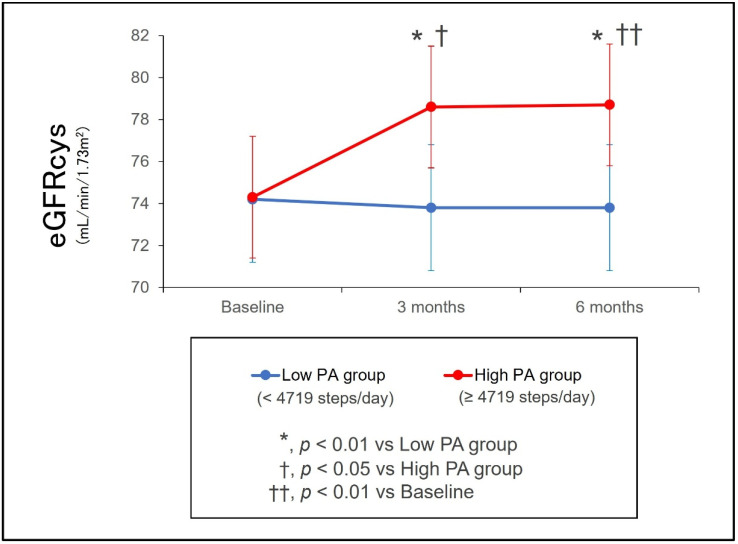
Longitudinal changes in the eGFRcys during the follow-up period (based on [54]). Data are presented as estimated marginal means, and the error bar is presented as the 95% confidence interval. eGFRcys, estimated glomerular filtration rate calculated from serum cystatin C; PA, physical activity.

We previously reported that, in patients with acute coronary syndrome after hospital discharge, habitual physical activity quantified by daily step counts significantly influenced eGFRcys trajectory [53,54,55,56]. When patients were classified into high-activity and low-activity groups, eGFRcys over 3 months declined by −2.9 mL/min/1.73 m^2^ in the low-activity group but increased by +6.7 mL/min/1.73 m^2^ in the high-activity group, demonstrating not only attenuation of decline but measurable improvement [53]. This association persisted at 6 months (Figure 2). Sensitivity analyses stratified by CKD status also demonstrated a significant positive correlation between daily step counts and changes in eGFRcys, confirming a dose–response relationship between higher physical activity and improved kidney function [54]. These findings suggest that structured support and education promoting physical activity after AMI are associated with more favorable eGFRcys trajectories; however, residual confounding cannot be excluded, and prospective trials are needed to determine whether activity promotion causally modifies CKD progression or incident CKD risk.

To define clinically relevant physical activity targets, we further examined step count thresholds and exercise intensity associated with improved renal outcomes. Maintaining an average of ≥5186 steps/day [55] and performing moderate-to-vigorous physical activity (≥3 metabolic equivalents) for ≥30 min at a frequency of ≥3 days/week [56] were associated with improvement in eGFRcys in patients with AMI. These studies uniquely define quantitative thresholds for activity volume, intensity, and frequency associated with renal benefit. Such targets may serve as practical minimum recommendations for patients with overlapping cardiac and renal dysfunction who aim to preserve kidney function during recovery.

## 5. Significance of Cystatin C Assessment When Evaluating Renal Endpoints

Interpretation of renal outcomes during rehabilitation requires careful consideration. Improvements in exercise capacity and nutritional status can increase creatinine generation, thereby confounding eGFRcreat. This limitation is particularly relevant in rehabilitation settings, where gains in muscle mass and physical conditioning may influence eGFRcreat independently of true changes in glomerular filtration. The 2024 Kidney Disease: Improving Global Outcomes guideline recommends appropriate assessment of kidney function, including eGFRcys, when creatinine-based estimates are potentially inaccurate. The guideline also recommends confirming kidney function using combined creatinine–cystatin C equations [57].

Hama et al. demonstrated that attenuation of eGFR decline associated with CR was more clearly detected using eGFRcys than eGFRcreat [47]. In our observational study evaluating physical activity after AMI, associations between activity levels and kidney function trajectories varied according to whether GFR was estimated using cystatin C or creatinine [53]. Collectively, these findings support prioritizing cystatin C-based equations, or combined equations, in studies examining renal trajectories during exercise interventions, particularly when changes in muscle mass are anticipated.

## 6. Mechanistic Pathways and Translational Evidence for CR-Mediated Renal Protection

Growing mechanistic evidence supports the biological plausibility of exercise-based interventions in CKD and cardiorenal conditions. Exercise favorably modulates endothelial function, oxidative stress, inflammation, insulin sensitivity, and skeletal muscle mitochondrial function [58]. Experimental data indicate that aerobic training upregulates Klotho expression and attenuates renal fibrosis in aging models [59], although extrapolation to clinically complex, multimorbid populations warrants caution. Conceptually, exercise functions as a multi-target intervention that concurrently influences interconnected pathways driving CKD progression and cardiovascular risk [60].

In CRS/CKM conditions, endothelial dysfunction, impaired perfusion, neurohormonal activation, metabolic dysregulation, vascular stiffness, and endocrine abnormalities converge. Exercise likely exerts synergistic rather than isolated effects. A central mechanism involves nitric oxide (NO) signaling and renal microvascular integrity. In experimental chronic heart failure with CRS, exercise increased renal nitric oxide synthase activity and upregulated endothelial and neuronal isoforms (eNOS/nNOS), accompanied by improved creatinine clearance and reduced urinary albumin excretion [61]. Complementary hypertension models demonstrated enhanced renal eNOS/nNOS expression and nitrate/nitrite availability, with concurrent reductions in albuminuria and oxidative stress markers [62]. These adaptations provide a coherent mechanistic link between improved cardiovascular physiology and the preservation of renal microcirculation.

Oxidative stress and chronic inflammation, key drivers of CKD progression and muscle–kidney and heart–kidney interactions, represent additional therapeutic targets. In salt-sensitive hypertension models, exercise reduced renal oxidative stress, improved glomerulosclerosis, and attenuated renal injury markers, even without substantial blood pressure reduction [63]. In metabolic disease models relevant to CKM, chronic aerobic training mitigated early diabetic nephropathy, suppressed glycation intermediates and oxidative stress markers, and improved albuminuria and creatinine clearance [64]. These findings support a multi-target framework in which renal protection reflects concurrent attenuation of oxidative and metabolic stress and restoration of protective signaling pathways, including NO.

Neurohormonal regulation further integrates exercise into established cardiorenal therapeutic paradigms. Given the central role of renin–angiotensin–aldosterone system (RAAS) activation and sympathetic overactivity in CRS pathophysiology, it is notable that moderate exercise did not exacerbate renal injury in hypertensive renal failure models and reduced proteinuria and glomerulosclerosis. Moreover, exercise combined with pharmacologic RAAS blockade conferred incremental renoprotective effects [65]. Endocrine and mineral metabolism pathways may also contribute. Aging models demonstrate exercise-induced Klotho upregulation and reduced fibrosis-related signaling [59], and meta-analytic evidence in patients with CKD indicates increased circulating Klotho and decreased fibroblast growth factor 23 following exercise training [66], supporting endocrine cross-talk as a plausible mediator of renal benefit.

Vascular health represents an intermediate and modifiable determinant of renal outcomes. Systematic reviews indicate that exercise improves vascular function in patients with CKD, providing a mechanistic basis for enhanced renal microcirculation and reduced downstream injury. In CRS/CKM, characterized by atherosclerosis and endothelial dysfunction, these vascular adaptations may explain stabilization of albuminuria and renal trajectories observed in selected cohorts [67]. Collectively, these converging data support a systems-level model in which exercise-based rehabilitation modulates upstream determinants of kidney injury across endothelial, inflammatory, neurohormonal, metabolic, endocrine, and vascular domains, thereby strengthening resilience within the cardiorenal axis [68].

## 7. Prognostic Implications

Prognostic evidence for rehabilitation is most robust for functional capacity and hospitalization outcomes. Exercise-based CR reduces hospitalizations in patients with heart failure [27]. Whether comparable benefits extend to cardiovascular patients with substantial renal dysfunction warrants clarification. A retrospective cohort study of patients with cardiovascular disease and advanced CKD (eGFR < 30 mL/min/1.73 m^2^) demonstrated a significantly lower cumulative incidence of major cardiovascular events among CR participants compared with nonparticipants [69]. In patients receiving dialysis after coronary artery bypass grafting, CR participation was associated with improved survival, and cost-effectiveness analyses suggested economic value in high-risk populations [70,71]. Nevertheless, large randomized trials evaluating mortality in cardiorenal overlap populations remain absent.

## 8. Evidence Gaps and Future Directions

CR for patients with CRS may help preserve and improve physical function, renal function, and survival; however, the optimal exercise prescription remains undefined. Most available evidence is derived from conventional CR protocols developed for patients with cardiovascular disease and may not fully address the complex pathophysiology of cardiorenal multimorbidity. In this context, the optimal frequency, intensity, time, type, volume, and progression of exercise require clarification through adequately powered prospective clinical trials. Compared with conventional CR programs, renal-focused rehabilitation strategies may require greater emphasis on symptom-limited progression and closer clinical monitoring, particularly in patients with advanced CKD or CRS. Safety considerations, including blood pressure lability and volume-related symptoms, further support investigation of individualized exercise prescriptions, including lower-intensity or home-based training models implemented with appropriate supervision.

Integrating rehabilitation into routine care for patients with cardiorenal multimorbidity will likely require coordinated multidisciplinary care pathways aligning cardiology, nephrology, and rehabilitation services. Potential implementation strategies include shared referral criteria, unified risk stratification frameworks, and coordinated monitoring of symptoms, blood pressure, volume status, and renal biomarkers. In addition, common barriers to participation—such as frailty, multimorbidity, and limited access to facility-based programs—highlight the potential role of home-based, hybrid, and tele-rehabilitation approaches to enhance accessibility and long-term adherence [72,73,74].

Importantly, previous CR studies in cardiovascular populations that included renal endpoints have rarely evaluated clinically meaningful renal outcomes, such as eGFR trajectory, longitudinal changes in albuminuria, or the incidence of acute kidney injury. Furthermore, most available studies have been small observational investigations, limiting causal inference. Accordingly, validation in large randomized controlled trials is needed. Future studies should also incorporate mechanism-based biomarkers—including NO-related indices, oxidative stress markers, and kidney injury biomarkers—to better elucidate biological pathways and facilitate precision-based personalization of exercise interventions in patients with CRS.

## 9. Conclusions

Rehabilitation constitutes a central therapeutic strategy for patients with combined cardiac and renal dysfunction. Across populations with cardiovascular disease and CKD, exercise-based interventions consistently improve physical function, enhance quality of life, and promote cardiometabolic health. Although definitive evidence for renoprotection and survival benefit in advanced cardiorenal multimorbidity remains incomplete, current data indicate that rehabilitation is safe, biologically plausible, and clinically meaningful. As the prevalence of cardiorenal multimorbidity increases, integration of structured rehabilitation into routine care, alongside guideline-directed pharmacotherapy and sustained support for long-term physical activity, offers a practical and patient-centered approach to improving functional resilience and overall outcomes. Future research should prioritize large randomized controlled trials evaluating functional and renal endpoints to clarify the clinical impact of rehabilitation in patients with cardiorenal multimorbidity.

## Figures and Tables

**Table 1:** Comparison of cardiorenal syndrome and cardiovascular–kidney–metabolic syndrome frameworks.

Domain	CRS	CKM Framework
Concept	Pathophysiologic syndrome describing bidirectional heart–kidney interactions	Population health framework integrating cardiovascular, kidney, and metabolic disease across a risk continuum
Primary focus	Heart–kidney cross-talk and acute or chronic organ dysfunction	Integrated cardiometabolic and kidney risk continuum with prevention-oriented staging
Disease scope	Primarily heart and kidney dysfunction	Cardiovascular disease, CKD, obesity, diabetes, and metabolic risk factors
Classification	Five subtypes based on primary organ involvement and time course (CRS Types 1–5)	Staged continuum from risk to clinical disease (Stages 0–4)
Pathophysiological emphasis	Hemodynamics, congestion, neurohormonal activation (RAAS/SNS), and inflammation	Excess or dysfunctional adiposity, insulin resistance, metabolic dysregulation, systemic inflammation, and multisystem interactions
Clinical context	Often applied in acute or advanced settings (e.g., acute HF with AKI; advanced CKD–CVD interplay)	Designed for prevention, early risk identification, and long-term management across the life course
Clinical goal	Guide understanding and management of cardiorenal dysfunction	Coordinate prevention and treatment strategies across cardiovascular, renal, and metabolic health
Relevance to rehabilitation	Mechanistic rationale for exercise effects on hemodynamics, endothelial, and neurohormonal pathways	Emphasizes lifestyle interventions (physical activity and rehabilitation) across stages, supporting earlier and broader implementation

AKI, acute kidney injury; CKD, chronic kidney disease; CKM, cardiovascular–kidney–metabolic; CRS, cardiorenal syndrome; CVD, cardiovascular disease; HF, heart failure; RAAS, renin–angiotensin–aldosterone system; SNS, sympathetic nervous system.

**Table 2:** Studies evaluating renal outcomes of cardiac rehabilitation in cardiovascular populations.

Study (Year)	Population (Participants)	Design/Setting	Intervention/Exposure	Duration/Follow-Up	Renal Markers and Other Outcomes	Main Renal Finding(s)
Toyama et al. [42]	CVD + CKD (*n* = 19; exercise *n* = 10 vs. non-exercise *n* = 9)	Non-RCT; comparative (CR vs. non-CR)	CR vs. non-CR	12 weeks	eGFR (creatinine-based); lipids; AT-$\dot{V}$O_2_	CR: eGFR improved; ΔeGFR correlated positively with ΔAT-$\dot{V}$O_2_ and ΔHDL-C and negatively with Δtriglycerides.
Takaya et al. [43]	AMI (total *n* = 528; CKD subgroup *n* = 180)	Non-RCT; pre–post within CR participants; attendance stratification	3-month CR; CKD attendance: non-active (≤1/wk) vs. active (≥1.1/wk)	3 months	eGFR (creatinine-based); peak $\dot{V}$O_2_; BNP	CKD subgroup: eGFR improved 48 ± 12 → 53 ± 15 (*p* < 0.001); active CKD improved 50 ± 10 → 53 ± 13; non-active no change; non-CKD no change.
Kimura et al. [44]	Male CVD patients without macroalbuminuria (*n* = 98); CR *n* = 23 vs. non-CR *n* = 75	Non-RCT; retrospective observational	CR (1–3/wk + home exercise) vs. non-CR	6 months	Urinary ACR; eGFR	CR: ACR decreased 43 ± 71 → 17 ± 20 mg/g (*p* < 0.05); eGFR unchanged. Non-CR: eGFR decreased 72 ± 18 → 67 ± 17 (*p* < 0.001).
Iso et al. [45]	CR participants; *n* = 67 (non-CKD *n* = 34; mild CKD *n* = 14; mod–severe CKD *n* = 19)	Non-RCT; retrospective	CR: supervised aerobic 1–2 × /wk + daily home walking	5 months (plus renal re-evaluation 3 months post program)	eGFR (JMDRD; creatinine-based)	Mod–severe CKD group: eGFR 40.8 ± 7.4 → 43.2 ± 12.6 (not significant); age inversely associated with eGFR change; <70 y showed significant eGFR increase.
Fujimi et al. [46]	CVD; *n* = 49 (CR group *n* = 23 vs. non-CR *n* = 26)	Non-RCT; comparative (CR vs. Non-CR)	1-year CR program vs. non-CR	1 year	eGFR (creatinine-based); UN/Cr/K/Hct	Overall: no significant changes in eGFR in either group; in the CR group, low baseline eGFR subgroup (<51) showed significant eGFR increase after 1 year.
Hama et al. [47]	CVD with CKD; *n* = 86	Non-RCT; single-arm	3-month CR program	3 months	eGFRcys (primary); eGFRcreat	eGFRcys improved 45.2 ± 11 → 47.3 ± 13 (*p* = 0.023); eGFRcreat unchanged.
Kitajima et al. [48]	Elderly CVD outpatients >65 y; *n* = 88	Non-RCT; longitudinal cohort	Long-term outpatient CR follow-up	Up to 5 years	eGFR (creatinine-based); AT; LVEF; BNP	eGFR, AT, LVEF, and BNP were maintained over 5 years; no significant change vs. baseline reported.
Sasamoto et al. [49]	CVD; *n* = 136 (≥75 y subgroup *n* = 55)	Non-RCT; prospective intervention (CR vs. non-CR)	CR and physical activity promotion	3 months	eGFRcys	ΔeGFRcys: non-CR −2.27 vs. CR +0.48 mL/min/1.73 m^2^ (*p* = 0.022); ≥75 y: −3.83 vs. −1.08 (*p* = 0.039).
Hama et al. [50]	CVD with CKD (15 ≤ eGFRcys < 60); *n* = 203 (G3a *n* = 122; G3b *n* = 60; G4 *n* = 21)	Non-RCT; retrospective cohort	CR program (pre vs. post) with baseline-stage stratification	3 months	eGFRcys	eGFRcys improvement: +1.3 (G3a), +3.1 (G3b), +4.8 (G4) mL/min/1.73 m^2^; lower baseline eGFRcys associated with greater %ΔeGFRcys.

CVD, cardiovascular disease; CKD, chronic kidney disease; CR, cardiac rehabilitation; RCT, randomized controlled trial; AMI, acute myocardial infarction; eGFR, estimated glomerular filtration rate; eGFRcreat, creatinine-based estimated glomerular filtration rate; eGFRcys, cystatin C–based estimated glomerular filtration rate; JMDRD, Japanese Modification of Diet in Renal Disease equation; ACR, albumin-to-creatinine ratio; AT-$\dot{V}$O_2_, oxygen uptake at anaerobic threshold; peak $\dot{V}$O_2_, peak oxygen uptake; BNP, B-type natriuretic peptide; HDL-C, high-density lipoprotein cholesterol; UN, urea nitrogen; Cr, creatinine; K, potassium; Hct, hematocrit; LVEF, left ventricular ejection fraction.

## Data Availability

This narrative review is based solely on previously published studies and publicly available sources. No new data was generated, and therefore data sharing is not applicable.

## Abbreviations

The following abbreviations are used in this manuscript:

ACR	Albumin-to-creatinine ratio
AHA	American Heart Association
AMI	Acute myocardial infarction
AT	Anaerobic threshold
BNP	B-type natriuretic peptide
CKD	Chronic kidney disease
CKM	Cardiovascular–kidney–metabolic
CR	Cardiac rehabilitation
Cr	Creatinine
CRS	Cardiorenal syndrome
CVD	Cardiovascular disease
eGFR	Estimated glomerular filtration rate
eGFRcreat	Creatinine-based estimated glomerular filtration rate
eGFRcys	Cystatin C-based estimated glomerular filtration rate
eNOS	Endothelial nitric oxide synthase
FGF23	Fibroblast growth factor 23
Hct	Hematocrit
HDL-C	High-density lipoprotein cholesterol
JMDRD	Japanese Modification of Diet in Renal Disease equation
K	Potassium
KDIGO	Kidney Disease: Improving Global Outcomes
LVEF	Left ventricular ejection fraction
NO	Nitric oxide
nNOS	Neuronal nitric oxide synthase
Peak VO_2_	Peak oxygen uptake
RAAS	Renin–angiotensin–aldosterone system
RCT	Randomized controlled trial
UN	Urea nitrogen
